# Potential bias of daily soil CO_2_ efflux estimates due to sampling time

**DOI:** 10.1038/s41598-017-11849-y

**Published:** 2017-09-20

**Authors:** Alejandro Cueva, Stephen H. Bullock, Eulogio López-Reyes, Rodrigo Vargas

**Affiliations:** 1 Departamento de Biología de la Conservación, Centro de Investigación Científica y de Educación Superior de Ensenada, B.C., Ensenada, Baja California 22860 Mexico; 2 Department of Plant and Soil Sciences, University of Delaware, Newark, DE 19716 USA

## Abstract

Soil respiration (Rs) has been usually measured during daylight hours using manual chambers. This approach assumes that measurements made during a typical time interval (e.g., 9 to 11 am) represent the mean daily value; locally, this may not always be correct and could result in systematic bias of daily and annual Rs budgets. We propose a simple method, based on the temporal stability concept, to determine the most appropriate time of the day for manual measurements to capture a representative mean daily Rs value. We introduce a correction factor to adjust for biases due to non-optimally timed sampling. This approach was tested in a semiarid shrubland using 24 hr campaigns using two treatments: trenched plots and plots with shrubs. In general, we found optimum times were at night and potential biases ranged from −29 to + 40% in relation to the 24 hr mean of Rs, especially in trenched plots. The degree of bias varied between treatments and seasons, having a greater influence during the wet season when efflux was high than during the dry season when efflux was low. This study proposes a framework for improving local Rs estimates that informs how to decrease temporal uncertainties in upscaling to the annual total.

## Introduction

Soil respiration (Rs) represents the second largest flux within the terrestrial carbon cycle, being surpassed only by gross primary productivity^1^. This flux is estimated to be an order of magnitude greater than the CO_2_ input to the atmosphere from anthropogenic fossil fuel combustion^2^. Rs represents a net loss of carbon derived from root respiration and from microbial metabolism of soil carbon^3,4^, the largest carbon pool globally^5^. Rs has complex spatio-temporal biophysical controls that vary on different scales^6^ as a consequence of changes in biotic (e.g., photosynthesis^7–10^, microbial community^11^) and abiotic (e.g., soil temperature^12,13^, soil moisture^14,15^, soil texture^16^) factors. It is important to recognize that a small change within this pool could represent a significant feedback to the earth system^17^. Thus, sampling schemes and measurement strategies should be discussed to improve reports of Rs at the site level and across the world.

Rs is a composite of two main sources, heterotrophic (e.g., microbial metabolism) and autotrophic (root and mycorrhizae respiration)^4^. Partitioning of those sources is commonly done using trenching experiments^18^, where roots are excised and excluded from small plots so that microbial metabolism can be assumed to be the only source of Rs. Understanding the contributions of autotrophic and heterotrophic respiration is important because they may respond differently to temperature, with different temporal correlations on a variety of time scales^19^.

**Figure 1:**
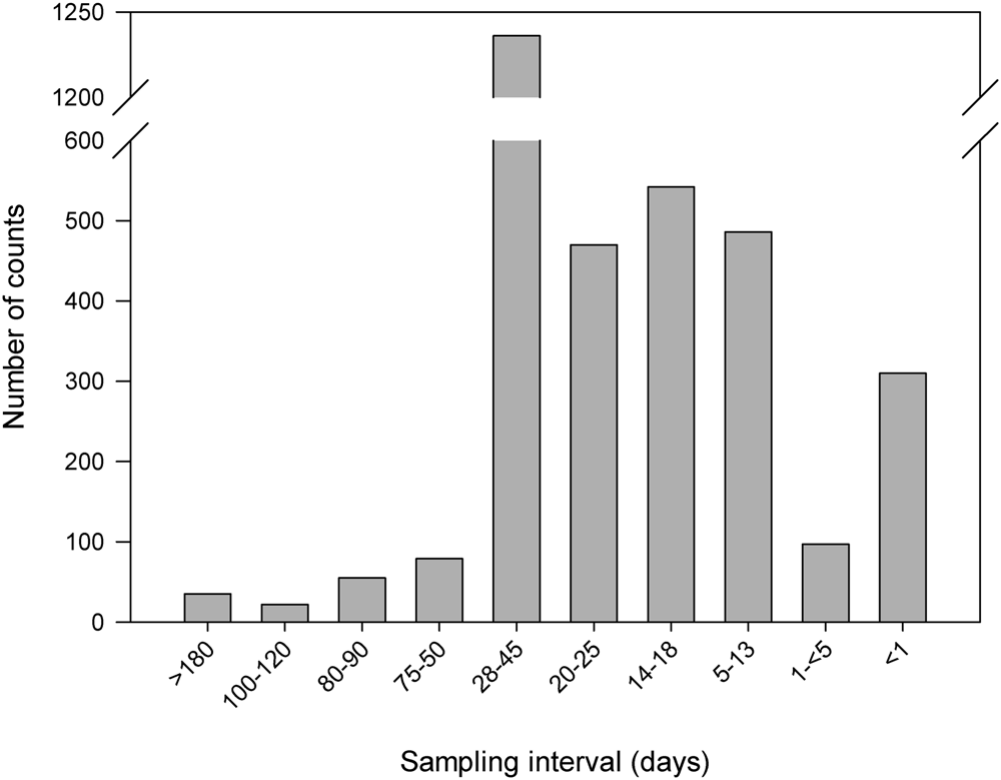
Histogram of number of entries sorted by sampling interval reported in the Soil Respiration Database (SRDB V3.0)^24^. Note that the most common sampling interval is from 28–45 days (e.g., monthly, n = 1236), followed by 14–18 (e.g., biweekly, n = 542). Also note that, despite the sampling interval, the annual coverage could be less than 365 days. The total number of entries in the SRDB V3.0 is 5174, but only 3332 reported a sampling interval. The SRDB V3.0 has data from 1961 to 2011.

Rs has been measured for almost 90 years^20^ and commonly has been measured using non-steady-state, manually-initiated portable chambers. Manual measurements have been popular around the world because of their portability, low implementation costs, and fewer power and security issues. Measurements using manual chambers are rapid (samples obtained within minutes), object-oriented (looking for differences between treatments without limits to their distribution), and involve visual assessment of the sample unit for every measurement. Results from these manual measurements have relatively good information of spatial variability (due to easy implementation) and are usually integrated to estimate longer-period emissions^21^. These temporal integrations include annual fluxes, although this derives from a record with temporal gaps^22^ due to the low frequency of sampling typical of manual chambers (Fig. 1). Furthermore, measurement campaigns are commonly done in daylight hours, and assume that measurements made at a specific time interval (e.g., 9 to 11 am) represent the mean daily value. Locally, that assumption could cause systematic under- or over-estimation and contribute to bias or error in annual estimates from local to global scales (see Barton, *et al*.^21^ for an example of N_2_O fluxes and Vargas and Allen^23^ for CO_2_ fluxes).

Individual efforts to monitor Rs at the local scale are commonly performed in order to understand the temporal and spatial biophysical controls of Rs, as well as to estimate seasonal to annual carbon budgets. Those local results may be collected and input to databases of Rs for estimating global carbon budgets. However, any error in the measurements of Rs at the local scale could be propagated to the global estimation. Thus, it is important to obtain the best possible estimates at the local scale, in order to decrease uncertainties for upscaling purposes. For instance, the Global Soil Respiration Database^24^ has been constructed mostly from manual measurements of Rs. Remarkably, despite the long history of Rs measurements, little attention has been paid to how the sampling time during 24 hr influences the estimation of Rs, while other shortcomings and pitfalls of sampling have been addressed (e.g., systematic^25,26^ and random^27,28^ errors in instrument measurements, sample size and strategy^29–31^).

The present work addresses the need to determine the effect of sampling time on Rs measurements. We based our analysis on the temporal stability concept^32,33^. Rs for each hour has a relative difference (RD) with the 24 hr site-level mean Rs. In turn, these values of RD may be relatively stable across hours for some months (e.g., seasonally) and can be represented by their mean (MRD). Then, MRD values close to zero indicate sampling times that are optimum, being closest to the 24 hr mean; the concept can also be applied using the standard deviation or other moment. We performed our analysis on 24 hr Rs data from two treatments intended to separate heterotrophic and autotrophic respiration in a Mediterranean-climate shrubland. Our purpose was to determine the time at which Rs measurements are most representative of the daily mean value and how the estimate of annual Rs could be affected by this artifact. Here we also introduce a correction factor to address the possibility of adjusting Rs measurements that are less representative due to sampling time.

## Results and Discussion

**Figure 2:**
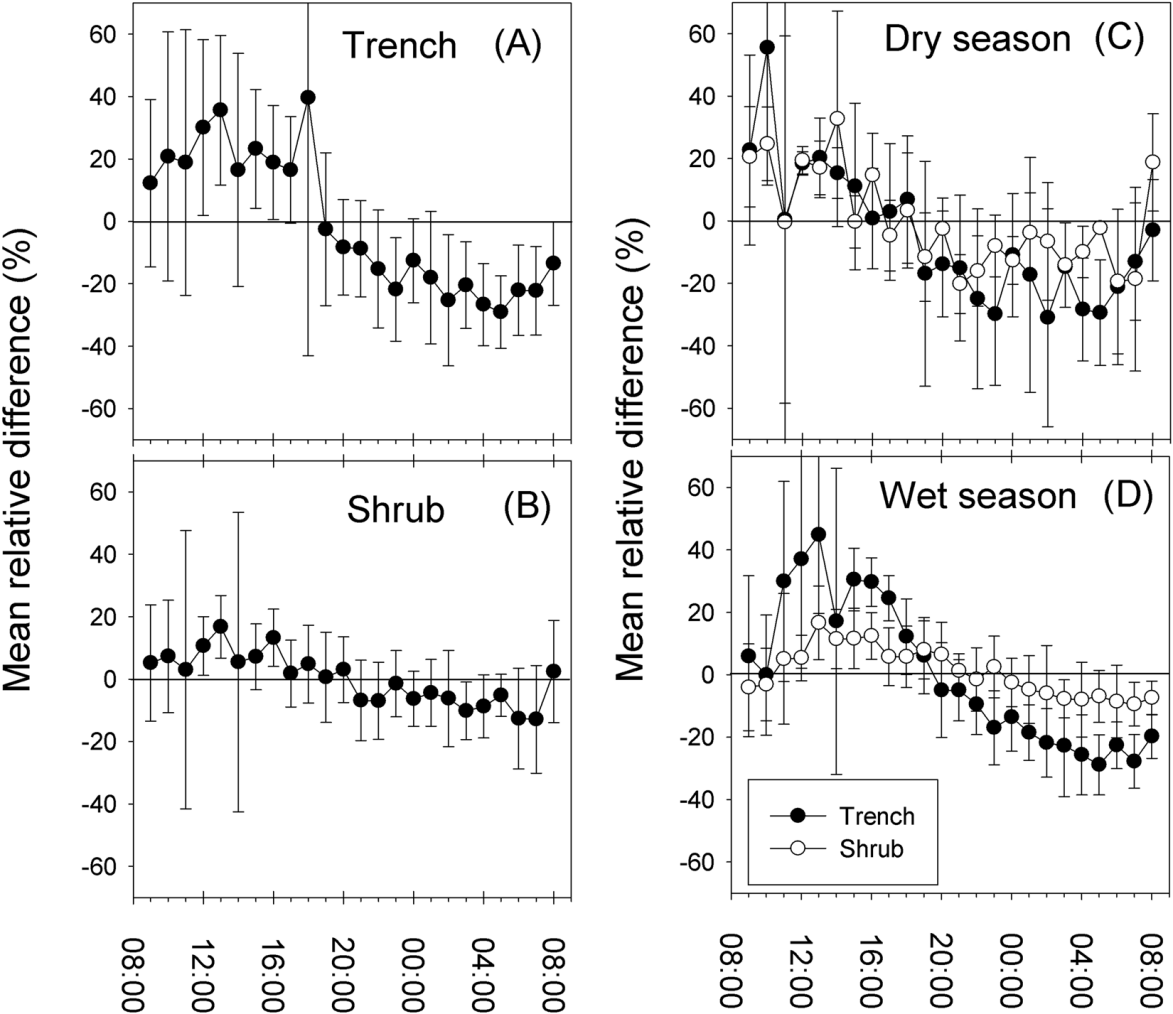
Mean relative difference (MRD) values ± standard deviation for all the 24 hr campaigns for the treatments (**A**) Trenched and (**B**) Shrub, and separated in (**C**) dry season and (**D**) wet season.

Using the temporal stability framework, we found that mean relative difference (MRD) values showed biases from −13 to + 17% in the shrub treatment, and from −29 to + 40% in the trench treatment (Fig. 2; Table 1). During daylight hours (e.g., from 8:00 to 19:00) measurements frequently over-estimated Rs in relation to the daily mean value of both trench and shrub treatments.

**Table 1:** Summary statistics for mean relative difference (MRD) values.

Treatment/Season	$\widetilde{\mu }$ (%)	σ (%)	Min (%)	Max (%)
Trench-All data	−0.08	22.1	−29.1	39.6
Shrub-All data	1.23	8.1	−12.9	16.8
Trench-Dry season	−11.99	21.2	−31.02	55.6
Shrub-Dry season	−3.14	15.4	−20.22	32.7
Trench-Wet season	−4.95	23.2	−28.9	44.9
Shrub-Wet season	−0.1	7.8	−9.5	16.6

$\widetilde{\mu }$: median; σ: standard deviation; Min: minimum value; Max: maximum value.

In general, we found that MRD values in the trench treatment had higher variability (−0.08 ± 22.1%; median ± standard deviation) than the shrub treatment (1.23 ± 8.1%; Fig. 2; Table 1). Furthermore, Rs in the trench treatment was often under-estimated: MRD median values were not close to zero because most hours were below the daily average (Table 1; Fig. 2). There was usually a greater negative bias during the dry season (−12% for trench and −3.14% for shrub) than during the wet season (−4.95% for trench and −0.1% for shrub) (Table 1).

The most appropriate time intervals for measuring Rs at our study site were not in the customary morning hours but rather from 17:00 to 19:00 in the shrub treatment and 20:00 to 21:00 in the trench treatment (Fig. 2). This could be due to Rs having a diurnal cycle, with its lowest values before sunrise, increasing through the morning and then decreasing more slowly sometime after noon and into the night. However, it has to be noted that the diurnal cycle of Rs did not follow a sinusoidal trajectory (as has been suggested for N_2_O fluxes^34^) in which case there would be two non-consecutive ideal hours that represent the mean daily value of Rs. Also, the hours with lowest MRD did not match those with the lowest SDMRD. Thus, the “ideal” hour may present difficulties both in terms of concepts (accuracy of estimates of the annual budget) and logistics (sampling near noon or midnight); thus, other criteria for choosing the sampling hours may have to be taken into account. Furthermore, we found differences when we evaluated the most appropriate time intervals for the dry and wet seasons. For example, during the dry season there was a more irregular pattern than during the wet season, such that appropriate hours for sampling Rs were dispersed across the 24 hr (Fig. 2). During the wet season, in contrast, the most appropriate hours to measure Rs were easily identifiable and consecutive (consecutive assigned ranks), ranging from 20:00 to 22:00 in the trench treatment and from 21:00 to 00:00 in the shrub treatment.

It is likely that optimal hours derived from the methodology we tested will vary among sites. For example, Davidson, *et al*.^25^ suggested that in a temperate mixed-hardwood forest the diel bias was ± 25% of the daily mean, the most adequate hours to measure Rs being in the mid-morning. In a young poplar forest, Gana, *et al*.^35^ found that the average of measurements made from 6:00 to 12:00 and 16:30 to 22:30 could represent the daily mean value of Rs, with potential biases of ± 20%. Moreover, in a temperate rainforest, Perez-Quezada, *et al*.^36^ found that daytime measurements of Rs always overestimated the Rs mean daily value derived from 24 h high-frequency measurements. Thus, the sampling times to obtain a representative daily Rs are likely to depend on the ecosystem or conditions studied, and should be determined for each site and season. Of course, study conditions include manipulations as in flux-partitioning experiments: our results showed that trenched plots had higher temporal bias and different optimal timing.

**Figure 3:**
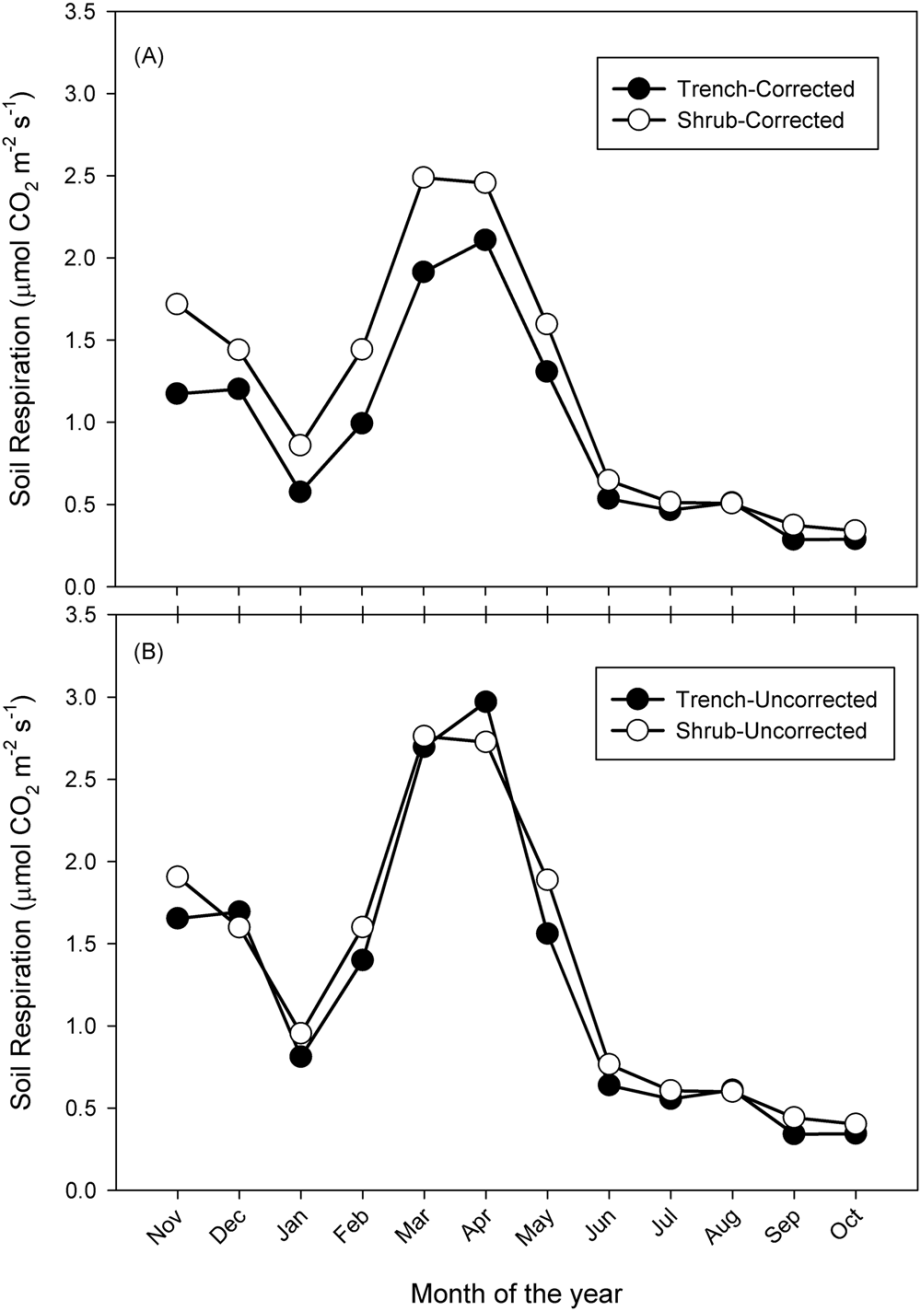
Corrected (**A**) and uncorrected (**B**) annual series of soil respiration. Note that we use a hydrological year (from November to October) instead of a calendar year (January to December).

When we applied the constant offset (Equation 4 in the Material and Methods Section; specific constant offsets for the dry and wet season) to our dataset, we found significant differences (Bayes Factor >3) between corrected and uncorrected estimations of Rs at the annual scale, as well as during the dry and wet season (Table 2; Fig. 3). When we compared the annual uncorrected values between the trench and the shrub treatments we did not find significant differences (Bayes Factor = 1.04; Supplementary Table 1). However, the contrast of the annual corrected values of Rs between the shrub and the trench treatments was significant (Bayes Factor = 37.41; Supplementary Table 1). Strong differences were always found during the wet season (Bayes Factor >3; Supplementary Table 1), but not during the dry season for either corrected or uncorrected values (Supplementary Table 1). This suggests that during the season of low ecosystem metabolic activity, together with an irregular pattern of the most representative sampling hours, the sampling time was not substantially influencing the estimations of Rs. However, during the wet season, sampling time influenced the estimations of both trenched and shrub treatments. We found that our previous system of sampling around midday could be over-estimating Rs by approximately 11 to 25% at the annual scale and by 10 to 30% during the wet season (Table 2).

**Table 2:** Annual and seasonal average (µ) ± standard deviation (σ) of soil respiration from monthly mid-day measurements in trench and shrub treatments, corrected and uncorrected for temporal bias.

Treatment	Season	µ (µmol CO_2_ m^−2^ s^−1^)	σ (µmol CO_2_ m^−2^ s^−1^)	Difference (%)	Bayes Factor*
Shrub–corrected	All year	1.20	0.77	−11.1	582.1
Shrub–uncorrected	All year	1.35	0.85		
Trench–corrected	All year	0.95	0.61	−25.2	22.08
Trench–uncorrected	All year	1.27	0.89		
Shrub–corrected	Dry season	0.66	0.47	−15.4	4.41
Shrub–uncorrected	Dry season	0.78	0.56		
Trench–corrected	Dry season	0.57	0.38	−14.9	5.17
Trench–uncorrected	Dry season	0.67	0.45		
Shrub–corrected	Wet season	1.73	0.64	−9.9	36.03
Shrub–uncorrected	Wet season	1.92	0.71		
Trench–corrected	Wet season	1.32	0.58	−29.4	20.07
Trench–uncorrected	Wet season	1.87	0.81		

^*^Values of Bayes Factor >1 indicate that data are *n* times better supported by the alternative hypothesis than by the null hypothesis.

When we compared the relationships of Rs with its main drivers (i.e., soil temperature and soil moisture), we did not find significant differences between corrected and uncorrected values of Rs at seasonal or annual scales (95% confidence intervals; Supplementary Table 2). Also, the variance explained remained similar between corrected and uncorrected values (Supplementary Table 2). Thus, functional relationships of Rs were not affected by correcting suboptimal estimates of mean Rs by a constant offset, although there were important effects on the estimates of seasonal and annual Rs.

## Conclusion

Our findings show that measurement of Rs in the customary morning to midday hours may not be appropriate for integrating temporal variability of Rs. At our study site, Rs measurements in daylight hours tend to overestimate the mean daily value of Rs, especially in the growing season. Repeated 24 hr campaigns can define sampling times that yield measurements most representative of the daily mean for each season. Such intensive but limited campaigns can also yield appropriate corrections for non-optimal timing of sampling in monitoring programs. It is noteworthy that the implications of this research may be geographically broad, and also may apply to other GHG emissions from soil (e.g., N_2_O, CH_4_), especially in treatment-effect experiments. For studies with manual systems, with one measurement per sampling position per sampling date, there should be baseline work on the 24 hr cycle, preferably per season, because convenient sampling could lead to over- or under- estimation of the annual Rs flux. It is important to combine locally appropriate timing of measurements with accurate spatial representation because these site-specific measurements provide information to databases that are used to estimate regional-to-global Rs.

## Material and Methods

### Estimation of the most representative time interval

Here we present a method to determine the most representative time interval to measure Rs, based on the temporal stability concept^32^. For a collection of sample positions where Rs is measured at nearly the same times over 24 hr, there is a stable relationship of the mean (or other statistic) for any time to the mean of the collection over all the times. This relationship may differ among the sample times and may also show seasonal changes. Then, the relative difference between an hourly mean of Rs and the daily mean Rs will have a range of values, and the closest to zero indicates the optimal time for sampling. This method could easily be applied regardless of site characteristics and to any periodic measurements, including other soil greenhouse gases.

The relative difference (RD) of Rs with respect to its expected value is given by:

1 $$R{D}_{j}=\frac{{x}_{ij}-\frac{{\sum }_{i=1}^{n}{x}_{ij}}{n}}{\frac{{\sum }_{i=1}^{n}{x}_{ij}}{n}},$$

where *j* represents the treatment (e.g., trench), *x* represents the Rs measurement at the *i*th time interval (e.g., 9:00 am), and *n* represents the number of intervals (e.g., *n* = 24 (hours) in a day). The RD values are specific for each 24 hr period. Thus, in order to determine a robust estimate of the most representative time interval, various 24 hr periods should be taken into account. Then, to integrate the RD of different 24 hr periods, the mean relative difference (MRD) is estimated as:

2 $$MR{D}_{i}=\frac{{\sum }_{N}^{N\,\max }R{D}_{i}}{N},$$

where *N* is the number of campaigns; we note that if *N* = 1 then MRD=RD. Thus, MRD values should range between −1 and 1, or may be multiplied by 100 to be expressed as percentage. The standard deviation of the MRD (SDMRD) is defined by:

3 $$SDMR{D}_{i}=\sqrt{\frac{{\sum }_{N}^{N\,\max }{(R{D}_{i}-MR{D}_{i})}^{2}}{n-1}}$$

The most representative sample interval should be that with MRD closest to zero (e.g., the minimum difference in relation with its daily mean value) and lowest SDMRD (e.g., the minimum variability in relation with its mean value)^37^. Thus, MRD quantifies the systematic bias of Rs at each sampling time, while SDMRD quantifies the precision of the bias. Finally, ranks are assigned in ascendant order to each MRD value (i.e., 1 is the lowest negative MRD and 24 is the highest positive MRD). Thus, we propose that the “ideal” time interval to measure Rs would be that with the middle ranking (12 or 13 for 24 hourly samples).

In order to adjust for sampling in “non-ideal” time intervals, a correction factor can be used^33^:

4 $${\widehat{Rs}}_{j}=\frac{R{s}_{i}}{1+MR{D}_{i}},$$

where $\hat{{{Rs}}_{j}}$ is the Rs_i_ measurement at time *i*th corrected by the offset derived from the MRD_i_ for time *i*th.

### Study site

El Mogor is a MexFlux^38^ site (MX-EMg) located within the Valle de Guadalupe, Baja California, México (32.02982 N, 116.60449 W, 409 m asl). The climate at El Mogor is semiarid Mediterranean, with warm-dry summers and cool-moist winters. Vegetation is a mixture of chaparral and less-sclerophyllous species. The site was severely burned in 1988 and has recovered to ~50% shrub cover. For further information about El Mogor, see previous publications^39–41^.

### Sampling design and measurements

In August 2011 we established three 1 × 1 m trenched plots, within the chaparral but lacking shrubs; we installed three PVC collars of 10 cm diameter within each plot. A trench of ~20 cm width and ~50 cm depth was excavated around each plot, lined with plastic sheeting (~1 mm thick) and backfilled. The excavation depth was decided on the basis of previous studies of the depth distribution of chaparral roots, which showed >85% of the roots were in the upper 40 cm of the soil profile^42,43^. Herbaceous plants were removed as necessary during the study period. In areas surrounding the trenched plots (<5 m) we inserted three more collars, placed within 50 cm of the main stem of a shrub. The total number of collars was 2 (treatments; herein *trench* and *shrub*) × 3 (plots) × 3 (collars) = 18. Measurements in the trench and shrub plots were initiated three months after trenching (November 2011), to minimize the influence of disturbance.

We performed eight 24 hr campaigns during 2014 (March, April, June, October, and November), 2015 (November), and 2016 (January and April). Those sampling campaigns represented the growing (wet) and non-growing (dry) seasons, with 4 campaigns per season. Measurements of Rs, soil moisture and soil temperature were made hourly from 9:00 of day T to 8:00 of day T + 1. Furthermore, we made monthly measurements of Rs, from 12:00 to 14:00 at 25 collars on a grid pattern across 0.125 ha, in order to estimate annual carbon loss via Rs.

Soil respiration was measured using a LI-8100 (LI-COR, Lincoln, NE, USA) and a 10 cm survey chamber (model 8100-102). Measurements of soil temperature and volumetric water content (Theta Probe, ML2x) were done at ~10 cm depth within 30 cm of the Rs chamber.

### Statistical analysis

Differences between means were tested using the inverse of Bayes factors (a value of Bayes Factor >1 indicates that data are *n* times better supported by the alternative hypothesis than by the null hypothesis^44^) for Student’s t-test. Furthermore, we used Bayesian linear regressions in order to test if functional responses of Rs with its main drivers (soil temperature and soil moisture) were maintained or affected by Equation 4. All statistical analyses were made in JASP (V0.8.0.0; available at https://jasp-stats.org/).

## Electronic supplementary material

Supplementary Material
