# Supplementary material for: Potential bias of daily soil CO2 efflux estimates due to sampling time
Source: Sci Rep. 2017 Sep 20;7:11925. doi: 10.1038/s41598-017-11849-y (PMC5607316; doi:10.1038/s41598-017-11849-y)
Supplement: Supplementary file 1 — Supplementary Material [file 41598_2017_11849_MOESM1_ESM.pdf]

Potential bias of daily soil CO_2 efflux estimates due to sampling time

Alejandro Cueva[1*], Stephen H. Bullock[1], Eulogio López-Reyes[1], Rodrigo Vargas[2]

Affiliations:
[1]Departamento de Biología de la Conservación, Centro de Investigación Científica y de Educación Superior de Ensenada, B.C., Ensenada, Baja California 22860, México
[2]Department of Plant and Soil Sciences, University of Delaware, Newark, DE 19716, USA

*Corresponding author:
Alejandro Cueva
Departamento de Biología de la Conservación
Centro de Investigación Científica y de Educación Superior de Ensenada, B.C.
Ensenada, Baja California 22860, México
Email: acueva@cicese.edu.mx

Supplementary Table 1. Bayesian paired samples Student's T-test of the time series of soil respiration data

T-test		Bayes Factor*
Shrub corrected – Shrub uncorrected	All year	582.1
Trench corrected – Trench uncorrected	All year	22.08
Shrub corrected – Trench corrected	All year	37.41
Shrub uncorrected – Trench uncorrected	All year	1.04
Shrub corrected – Shrub uncorrected	Dry season	4.41
Trench corrected – Trench uncorrected	Dry season	5.17
Shrub corrected – Trench corrected	Dry season	1.69
Shrub uncorrected – Trench uncorrected	Dry season	1.61
Shrub corrected – Shrub uncorrected	Wet season	36.03
Trench corrected – Trench uncorrected	Wet season	20.07
Shrub corrected – Trench corrected	Wet season	47.01
Shrub uncorrected – Trench uncorrected	Wet season	0.45

*Values of Bayes Factor >1 indicates that data are *n* times better supported by the alternative hypothesis than by the null hypothesis

Supplementary Table 2. Bayesian linear relationships of soil respiration with soil temperature (Temp) and soil moisture (SWC).

Treatment	Season	Temp	BF	R^2	SWC	BF*	R^2
Shrub-corrected	All-year	-0.05 (-0.1, 0.003)	1.58	0.3	6.54 (2.04, 11.05)	5.65	0.51
Shrub-uncorrected	All-year	-0.05 (-0.1, 0.006)	1.42	0.28	7.06 (2.0, 12.1)	4.88	0.49
Trench-corrected	All-year	-0.025 (-0.06, 0.01)	0.89	0.188	4.75 (0.98, 8.5)	3.44	0.44
Trench-uncorrected	All-year	-0.04 (-0.09, 0.01)	1.09	0.23	7.24 (2.04, 12.4)	4.83	0.49
Shrub-corrected	Dry season	-0.005 (-0.12, 0.11)	0.57	0.004	6.24 (-54.1, 66.7)	0.58	0.02
Shrub-uncorrected	Dry season	-0.006 (-0.15, 0.14)	0.57	0.004	7.4 (-64.0, 78.8)	0.58	0.02
Trench-corrected	Dry season	0.005 (-0.06, 0.07)	0.57	0.01	-9.87 (-68.5, 48.8)	0.6	0.05
Trench-uncorrected	Dry season	0.006 (-0.07, 0.09)	0.57	0.01	-11.8 (-81.8, 58.2)	0.6	0.05
Shrub-corrected	Wet season	0.19 (0.003, 0.37)	2.04	0.66	3.67 (-5.1, 12.5)	0.79	0.25
Shrub-uncorrected	Wet season	0.21 (0.003, 0.41)	2.04	0.67	4.1 (-5.7, 13.8)	0.79	0.25
Trench-corrected	Wet season	0.18 (0.04, 0.31)	3.15	0.77	3.05 (-5.03, 11.1)	0.75	0.22
Trench-uncorrected	Wet season	0.25 (0.06, 0.44)	3.13	0.77	4.3 (-7.1, 15.7)	0.75	0.22

*Values of Bayes Factor (BF) >1 indicates that data are *n* times better supported by the alternative hypothesis than by the null hypothesis
